# Function and mechanism of the human SOD2 gene in mice cerebral ischemia/ reperfusion injury

**DOI:** 10.1590/acb393124

**Published:** 2024-08-05

**Authors:** Xitong Yang, Guangming Wang

**Affiliations:** 1 Dali University – The First Affiliated Hospital – Dali, Yunnan – China

**Keywords:** Transgenic, Ischemic Stroke, Manganese Superoxide Dismutase, Neuroprotection, Cerebrovascular Circulation

## Abstract

**Purpose:**

To investigate the neuroprotective effects of the SOD2 gene in cerebral ischemia reperfusion injury function and the underlying mechanisms in a mice model of middle cerebral artery ischemia reperfusion.

**Methods:**

SOD2 transgenic mice were engineered using transcription activator-like effector nucleases, and the genotype was identified using PCR after every three generations. Transgenic and C57BL/6J wild type mice were simultaneously subjected to the middle cerebral artery occlusion model.

**Results:**

SOD2 expression in the brain, heart, kidney, and skeletal muscle of transgenic mice was significantly higher than that in the wild type. Following ischemia reperfusion, the infarct volume of wild type mice decreased after treatment with fenofibrate compared to the CMC group. Infarction volume in SOD2 transgenic mice after CMC and fenofibrate treatment was significantly reduced. The recovery of cerebral blood flow in wild type mice treated with fenofibrate was significantly enhanced compared with that in the CMC group.

**Conclusions:**

The expression of SOD2 in transgenic mice was significantly higher than that in wild type mice, the neuroprotective role of fenofibrate depends on an increase in SOD2 expression.

## Introduction

Stroke is one of the leading causes of death and disability worldwide, accounting for approximately 5.5 million deaths and 44 million disabilities each year^1^.This has been shown to be particularly burdensome to patients and society in low- and middle-income countries^2^. More than 80% of all stroke cases worldwide are attributable to ischemic stroke, which is caused by cerebral artery occlusion^3^. Considering the increasingly aging global population and high disability rate of stroke, further studies on stroke, especially those on ischemic stroke, are urgently needed. The pathological type and pathogenesis of ischemic stroke are intricate^4^. Cerebral ischemia/reperfusion (I/R) injury is a complex pathological process involving multiple mechanisms. Persistent ischemia and hypoxia in brain tissue can induce neuronal apoptosis, which is the main mechanism of infarct enlargement and delayed neuronal injury^5^. Following cerebral I/R, increased free radical production and damage of the antioxidant system is one of the important mechanisms leading to oxidative stress injury. Oxidative stress can activate mitochondria or nuclear apoptotic signaling pathways and may also damage DNA, proteins, or biofilm lipids, thereby leading to cell damage and death, and the occurrence of ischemia reperfusion injury. Therefore, activating the antioxidant system against oxidative stress is a key mechanism to reducing ischemia reperfusion injury^6^.

Fenofibrate is a selective peroxisome proliferator activated receptor (PPARα) agonist, which is mainly used for regulating blood lipids. In recent years, increasing number of studies have shown that PPARα agonists have anti-inflammatory and immunomodulatory effects. Studies have found that fenofibrate has an obvious protective effect in traumatic brain injury^7^, through significant reduction of brain tissue damage caused by focal cerebral ischemia^8^, and plays a neuroprotective role in cerebral ischemia. Fenofibrate has been shown to play a protective role in acute focal cerebral ischemia reperfusion injury in mice by upregulating the mRNA expression of PPARα and reducing the lipid peroxidation injury^9^. When fenofibrate reduces damage caused by cerebral ischemia the manganese superoxide dismutase (SOD2) expression is increased^10^. However, it is not clear whether the neuroprotective effect of fenofibrate is related to the high level of expression of SOD2. The SOD2 is a key antioxidant enzyme of mitochondrial, and an important component of the intracellular antioxidant system, it maintains the cellular oxidation/antioxidant balance by clearing away excess free radicals in the mitochondria. Studies have confirmed that SOD2 plays an important role in the protection of cerebral I/R injury, it is an important molecular defense mechanism of cells against cerebral I/R injury^11^. Therefore, in this study, we constructed a SOD2 transgenic mouse model and detected the expression of SOD2. Using the Middle Cerebral Artery Occlusion (MCAO), we investigate whether SOD2 plays a neuroprotective role in cerebral ischemia.

## Methods

### Animals and Chemicals

Female and male C57BL/6J SPF mice, aged 6-8 weeks, were purchased from the Hunan Silaike Jingda Laboratory Animal Co. Ltd. (Hunan Changsha); Certificate number: SCXK-2016 (Xiang)-000. The experiments were approved by the Biomedical Ethics Committee of the first affiliated hospital of Dali university and were conducted in accordance with the guidelines for animal experiments issued by the National Institutes of Health.

The mice were fed standard food particles and tap water and the indoor temperature was maintained at 20-25°C. The animals were housed in polypropylene cages, maintained under a 12-h light/dark cycle.

Carboxymethyl cellulose (CMC), 2,3,5-triphenyltetrazolium chloride (TTC), and fenofibrate were purchased from Sigma Aldrich (St Louis, MO, USA). The protein extraction kit was purchased from Thermo Scientific (Waltham, MA, USA), and the protein concentration assay kit was purchased from Bio-Rad (Richmond, CA, USA). All other chemicals were manufactured by Sigma Aldrich (St Louis, MO, USA).

### Construction of transgenic mice

**Figure 1:**
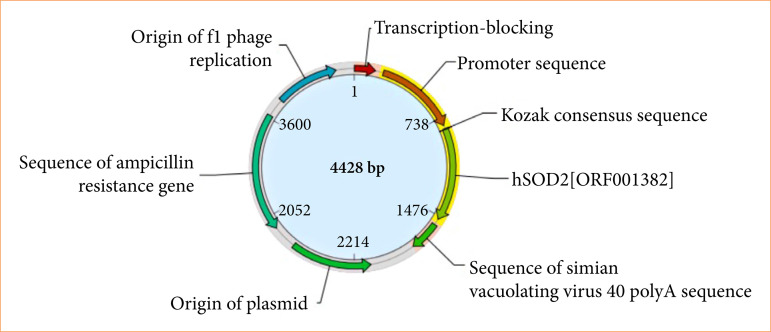
Carrier pRP (Exp) -CMV>hSOD2 atlas.

The SOD2 transgenic mouse model, with the background of C57BL/6J, was constructed by the Saiye (Guangzhou) biotechnology Co. Ltd. Center. To construct the transgenic mice, the target gene pRP (Exp) -CMV>hSOD2 carrying recombinant plasmid was injected into the male pronucleus of the oosperm by microinjection, followed by its transfer to the surrogate mother mouse to give birth naturally. The plasmid profile information of this experiment is shown in Fig. 1.

### PCR identification of genotypes in transgenic mice

The tail tissue of transgenic mice aged 6-8 weeks was taken, and genomic DNA was extracted by tissue genome extraction kit. Two pairs of primers were designed for genotype identification based on the sequence of the inserted target gene: the first pair of primers was the primer of the target gene with a length of 252 bp, whereas the second pair of primers was a reference primer with a length of 413 bp. The primer sequence is shown in Table 1. PCR amplification system (25 μL) was comprised of the following: ddH2O 8 μL, Premix Taq 12.5 μL, DNA 2.1 μL, product primer 0.8 μL, the upstream and downstream primers of the target gene were 0.4 μL each, and the upstream and downstream primers of the internal reference were 0.8 μL each. PCR reaction conditions were as follows: initial denaturation at 94°C for 30 min, followed by denaturation at 94 °C for 30 s, annealing at 66 °C for 35 s, with extension for 35 s at 72 °C; this cycle was repeated 38 times, and extension at 72 °C was carried out for 5 min in the last cycle. After the product was obtained, electrophoresis was performed with 1.5 % agarose gel. The results were observed in the gel imaging system after ethyl bromide staining.

**Table 1:** Two pairs of primer sequences used for genotype identification.

Primer	Primer sequences	Primer length
TGT Transgene PCR primer F	GTTTCAATAAGGAACGGGGACAC	252 bp
TGT Transgene PCR primer R	CCGGCCATCACCACTTTGTA	
TGI Internal control PCR primer F	ACTCCAAGGCCACTTATCACC	413 bp
TGI Internal control PCR primer R	ATTGTTACCAACTGGGACGACA	

### Animal group and drug treatment

Wild type and transgenic mice were randomly divided into 2 groups according to odd or even numbers: CMC group 8 mice were treated with CMC suspension at 10 mL 0.5 %/kg weight. Fenofibrate group 10 mice were treated with a dose of 30 mg/kg of fenofibrate according to the literature^12^. Fenofibrate was dissolved in CMC to form a suspension and mixed before administration by gavage. The two groups of mice received the drugs simultaneously, once a day, for three days. Surgery was performed within 1 h after the last dose.

### Middle cerebral artery occlusion^13^

The mice were anesthetized using 1.5 % isoflurane during surgery, and their rectal temperature was maintained at Origin of plasmid 37 ± 1°C using a heating pad. The right common and external carotid arteries were separated under direct visualization using a surgical microscope and ligated. A 8-0 nylon thread monofilament measuring 10 mm in length, coated with silicone resin and hardener mixture (Heraeus, Hanau, Germany), was inserted into the internal carotid artery via the common carotid to block the blood flow of the middle cerebral artery; the timing began when cerebral blood flow (CBF) dropped below 20 % of the original value. After 60 min, the nylon thread was removed to allow reperfusion. During the operation, CBF was monitored using a detector (BD Ltd, USA) diagnostics, which was placed on the surface of the cranium over the ischemic area, behind the margin of the left ear. After reperfusion for 30 min, a Laser Speckle image system was used to collect the images of CBF. The animals were humanely sacrificed 18 h after reperfusion, and the brain tissue was obtained for follow-up tests.

### Infarct volume measurement

Excessive isoflurane was used to sacrifice mice, and the brain was collected by separating it from the bone and meningeal membranes of the severed head; 5 coronal sections of 2 mm thickness were prepared from the brain tissue and stained with 2 % TTC for 30 min at room temperature. The infarct area was analyzed using the MCID image analysis system (Imaging Research, Inc., St Catherines, Ontario, Canada).

### Quantitative PCR measurements of SOD2 mRNA expression

Quantitative PCR measurements of SOD2 expression in strain with the largest number of breeding, tissues from the same four body parts (brain, heart, kidney, muscle) were taken from each of the transgenic and wild type mice. Total RNA was extracted from the tissue according to the Trizol instructions (Invitrogen), subsequently, cDNA was synthesized using a reverse transcription kit (Invitrogen) and PCR amplification was performed by iQ SYBR Green kit. The cycle conditions were as follows: initial denaturation at 95 °C for 2 min, followed by denaturation at 94 °C for 20 s, annealing at 60 °C for 20 s, with extension for 30 s at 73 °C; and extension once again for 5 min at 72°C. Once more, the cycle was repeated 40 times. The primers sequences of SOD2 included forward primer 5’- CAGACCTG CCTTACGACTATGG-3’ and reverse primer 5’- CTCGGTGGCGTTGAGATTGTT -3’; actin forward primer 5’-GACAGGATGCAGAAGGAGATTACT-3’, reverse primer TGATCCACATCTGCTGGAAGGT-3’.

### Western blotting

Selected transgenic and wild type mice, obtain tissue proteins from brain, heart, kidney and muscle. Total protein was extracted using the protein extraction kit, which detects the protein concentration using a Bio-Rad protein concentration assay kit. Sodium dodecyl sulfate-polyacrylamide gel electrophoresis was performed using 15 μg/5 µL of the protein (separation gel concentration: 8%), and was transferred to a polyvinylidene difluoride (PVDF) membrane by the Bio-Rad semi-dry transfer system. After washing with 0.1 M tris-buffered saline (TBS), 1% skim milk powder was added and incubated at room temperature for 30 min. Rabbit anti-mouse polyclonal (Cayman, Ann Arbor, MI, USA; 1:500), and goat anti-mouse antibody against β-actin monoclonal antibody (Sigma-Aldrich, St. Louis, MO, USA) (1:2000) were added and incubated with the membrane overnight at 4 °C. After washing thrice with 0.1 M TBS, the membrane was incubated with anti-goat or anti-rabbit secondary antibody (IRDye 800CW and IRDye 680CW label, respectively; 1:3500). After washing and scanning with the Bio-Rad western blot detection system, the images were analyzed using Image J (Image J 1.46r, Wayne Rasband, National Institute of Health, USA). The ratio of SOD2 to β-actin represents the gray value of SOD2 expression.

### Statistical analysis

All experimental data are presented as means ± standard deviation (SD). GraphPad Prism 5.00 for Windows (GraphPad Software, La Jolla California USA, www.graphpad.com) was used to analyze the data. The *p* < 0.05 were considered statistically significant.

## Results

### Identification of transgenic mice

**Figure 2:**
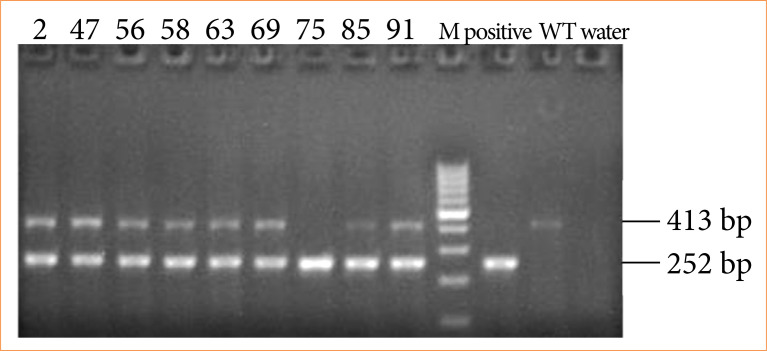
PCR identification results of SOD2 transgenic mice.

Every three generations of SOD2 transgenic mice were identified by PCR, and results were viewed by gel imaging (Fig. 2). Amplified bright bands at 413 bp and 252 bp demonstrated that SOD2 transgenic mice have a stable inheritance.

### SOD2 expression of transgenic mice

**Figure 3:**
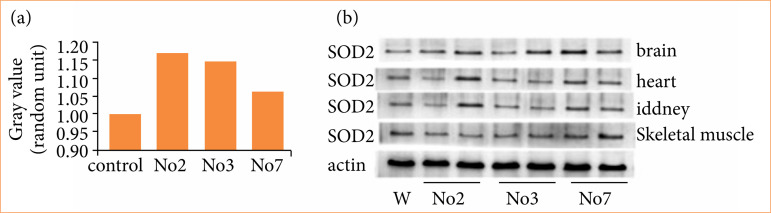
Identification of SOD2 transgenic mice. a. brain tissue expression of SOD2 in transgenic mice was detected by Q-PCR; b. Western blotting to detect the expression of SOD2 in different tissues of transgenic mice.

SOD2 transgenic mice interbred with C57BL/6J mice. We selected 2, 3, and 7 strains of transgenic mice with the most survival for western blotting and quantitative PCR (Q-PCR) detection. Image J software was used to perform signal strength analysis. Q-PCR results showed that mRNA expression level of SOD2 in brain tissue of SOD2 transgenic mice was significantly higher than in the control mice. Strains 2 and 3 were found to belong to highly expressed strains (Fig. 3a). Brain, heart, kidney, and skeletal muscle tissue protein levels were also significantly higher than in the control group (Fig. 3b). The results indicate that the expression of SOD2 in transgenic mice was significantly higher than in the wild type (*p* < 0.05).

### SOD2 transgenic mice infarct volume decreased during I/R

**Figure 4:**
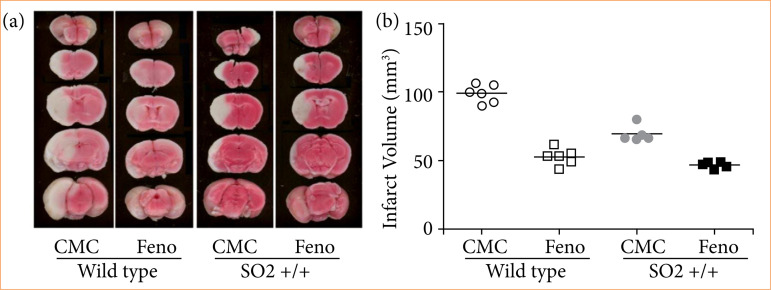
Fenofibrate pretreatment of SOD2 transgenic mice after I/R was performed with TTC staining.

Wild type and transgenic mice were prepared according to the MCAO model, with ischemia for 60 min followed by reperfusion for 90 min. The brain tissue was sliced and TTC staining was used to analyze infarct volume. The wild type CMC group showed obvious white infarcts, and the infarct volume was 99.068 ± 5.964 mm^3^, whereas the white infarcts of the wild type Fenofibrate group were significantly reduced (52.882 ± 5.427 mm^3^; Fig. 4a). T-test analysis showed that there was a significant difference in volume reduction (*p* = 0.000). The changes in body temperature and CBF in the two groups before and after perfusion are shown in Table 2; however, there was no statistical difference between the two groups (*p* > 0.05). Results from MCID imaging system showed that the infarct volume of transgenic SOD2 mice was significantly reduced compared to the wild type (Fig. 4b). After CMC treatment, the SOD2 transgenic mice infarct volume was 69.420 ± 5.132 mm^3^. The Feno group infarct volume was 46.801 ± 2.100 mm^3^. There was no statistical difference between the two groups of transgenic mice, that is, the neuroprotective effect of fenofibrate after cerebral I/R may be attributed to the high expression of SOD2. Increased SOD2 expression in SOD2 transgenic mice promotes the neuroprotective effect of fenofibrate on cerebral infarction.

**Table 2:** Rectal temperature (RT, °C) and regional cerebral blood flow (rCBF, %basal).

Monitoring indicators			Wild type			SOD2^+/+^ type	
			CMC (n = 6)	Feno (n = 6)		CMC (n = 5)	Feno (n = 5)
% Regional Cerebral Blood Flow	Before operation		100	100		100	100
	During the period of ischemia		10.51 ± 4.79	9.33 ± 3.53		9.45 ±3.19	10.58 ± 2.55
	reperfusion		99.47 ± 6.34	98.47 ± 5.62		99.32 ±9.69	101.24 ± 6.76
Body Temperature	Before operation		36.6 ± 0.59	36.2 ± 0.43		371. ± 0.96	36.4± 0.71
	During the period of ischemia		36.2 ± 0.10	36.3 ± 0.10		36.3 ± 0.08	36.3 ± 0.14
	reperfusion		36.3 ± 0.14	36.3 ± 0.08		36.4 ± 0.16	36.3 ± 0.32

### SOD2 transgenic mice CBF recovery after I/R injury

**Figure 5:**
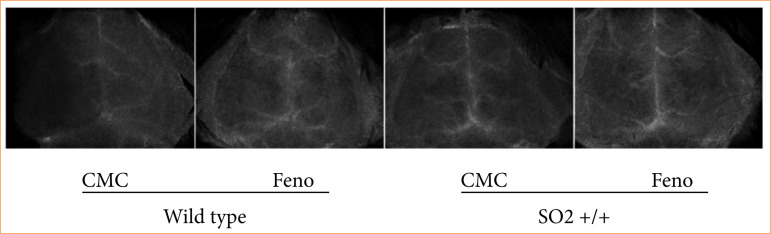
SOD2 transgenic mice CBF change after I/R.

Transgenic mice were prepared according to MCAO model, with ischemia for 60 min, reperfusion for 30 min, then by Laser Speckle image system to collect CBF pattern to monitor the changes of CBF. The results showed that Feno may restore CBF after I/R in wild type mice compared to the CMC group, and that the CBF was significantly enhanced. Feno was shown to promote CBF recovery after I/R in the wild type mice. Transgenic mice the CBF was enhanced,, and there was no significant difference in CBF between CMC and Feno groups (Fig. 5), thereby indicating that the neuroprotective role of Feno depends on the increase in SOD2 expression.

## Discussion

Transgenic animals are engineered from the insertion of exogenous genes into the genomes of animal germ cells by experimental methods, whereby the gene has an insertion characteristic and the animals can reproduce normally^14^. Transgenic animals have become a powerful tool to explore the mechanisms of gene regulation, carcino genesis, and immune system response^15^^,^ 16. In this study, SOD2 was used as the target gene to establish SOD2 transgenic mice by microinjection. PCR was used to identify stable inheritance. Transgenic mice were mated with C57BL/6J mice to produce offsprings. The progeny mice detected by quantitative fluorescence PCR and western blotting showed expression of SOD2 in transgenic mice, which was shown to be higher than in the wild type. Using the MCAO model, expression of SOD2 was directly proportional to the degree of neuroprotection.

Oxidative damage is considered to be one of the key factors in cerebral I/R injury, whereby the balance between the oxidation system and the antioxidant system is destroyed, and the body generates excessive reactive oxygen species (ROS), which causes membrane lipid peroxidation, thereby leading to pathological damage. SOD2 is the main antioxidant enzyme against excessive ROS in the body, which scavenges oxygen free radicals and protects the body from oxidative damage. SOD2 vitality level reflects the ability of scavenging to oxygen free radicals and antioxidant capacity^17^^,^ 18. The study results showed that the expression of SOD2 in transgenic mice was apparently higher than that in the wild type, and the neuroprotective of Fenofibrate in SOD2 transgenic mice was more distinct.

Previous studies have shown that Feno plays a protective role in cerebral I/R injury, activates PPARα, and inhibits NF-KB activity, antioxidant stress, and inhibits inflammation in the central nervous system^19^. Fenofibrate was also shown to have a protective effect in traumatic brain injury, and decrease the damage of brain tissue caused by focal cerebral ischemia^20^. In this study, wild type mice by Fenofibrate treatment after cerebral ischemia infarction volume was significantly reduced, Feno group CBF was restored after I/R. That is, after cerebral ischemia Feno can reduce cerebral infarction volume, restore CBF, and reduce brain damage in wild type mice. The neuroprotective effect was more remarkable in the Feno group of transgenic mice. The results demonstrate that the enhanced neuroprotective effect of Feno after cerebral ischemia in transgenic mice may be related to the increased expression of SOD2.

SOD2 is the oxygen free radical scavenging enzyme, which removes superoxide anion free radical, blocks the chain reaction of lipid peroxidation, and protects tissues from free radical damage. SOD2 activity reflect the change in antioxidant capacity^21^, an endogenous antioxidant defense mechanism that plays a role in the process of reducing and scavenging free radicals^22^. During I/R, a large number of free radicals are generated; however, the ability to clear away free radicals remains unchanged, as reperfusion tissue regains oxygen and relieves the ischemia and hypoxia conditions, which at the same time produce and accumulate a large number of free radicals and aggravate brain tissue damag^23^ . ROS are also involved in signaling, cell differentiation, and apoptosis, and affects the mechanism by which new cells and blood vessels form^24^. Activation of SOD2 also affects the proliferation and angiogenesis of vascular endothelial cells^25^^,^ 26. Previous studies have shown that after cerebral ischemia, SOD2 deficiency aggravates cerebral infarction, and expression of SOD2 is significantly decreased^27^. In this study, after I/R in transgenic mice, the CBF was significantly enhanced and expression of SOD2 increased in transgenic mice compared to the wild type. The neuroprotective role of Feno in transgenic mice may be attributed to increased expression of SOD2, which reduces ROS, relieves oxidative damage, enhances CBF, and alleviates brain damage to protect the nerve.

In this study, it was found that the increased expression of SOD2 is crucial to the injury and prognosis of cerebral ischemia reperfusion. These results may contribute to the clinical treatment of stroke; however, more research is needed to support these findings.

During the cerebral ischemia period SOD activity decreased. With the drug treatment, SOD and other antioxidant active enzymes recovery may help the treatment of cerebral ischemia. The study had some limitations: it was an animal experiment, and further studies are needed to confirm the results in humans. More studies are also needed to elucidate the underlying pathophysiology in terms of its cytology and molecular biology.

In conclusion, transgenic mice SOD2 expression was significantly higher than in the wild type. After cerebral ischemia, transgenic mice reduced the infarction volume, CBF was enhanced, and the relieved oxidative damage plays a neuroprotective role.

## Data Availability

All dataset were generated or analyzed in the current study
